# Genomic Analysis Reveals Contrasting PIFq Contribution to Diurnal Rhythmic Gene Expression in PIF-Induced and -Repressed Genes

**DOI:** 10.3389/fpls.2016.00962

**Published:** 2016-07-04

**Authors:** Guiomar Martin, Judit Soy, Elena Monte

**Affiliations:** Center for Research in Agricultural Genomics, CSIC-IRTA-UAB-UBBarcelona, Spain

**Keywords:** phytochrome-interacting factors PIFs, PIF-regulated transcriptional network, diurnal growth, *Arabidopsis*, short day, circadian clock

## Abstract

Members of the PIF quartet (PIFq; PIF1, PIF3, PIF4, and PIF5) collectively contribute to induce growth in *Arabidopsis* seedlings under short day (SD) conditions, specifically promoting elongation at dawn. Their action involves the direct regulation of growth-related and hormone-associated genes. However, a comprehensive definition of the PIFq-regulated transcriptome under SD is still lacking. We have recently shown that SD and free-running (LL) conditions correspond to “growth” and “no growth” conditions, respectively, correlating with greater abundance of PIF protein in SD. Here, we present a genomic analysis whereby we first define SD-regulated genes at dawn compared to LL in the wild type, followed by identification of those SD-regulated genes whose expression depends on the presence of PIFq. By using this sequential strategy, we have identified 349 PIF/SD-regulated genes, approximately 55% induced and 42% repressed by both SD and PIFq. Comparison with available databases indicates that PIF/SD-induced and PIF/SD-repressed sets are differently phased at dawn and mid-morning, respectively. In addition, we found that whereas rhythmicity of the PIF/SD-induced gene set is lost in LL, most PIF/SD-repressed genes keep their rhythmicity in LL, suggesting differential regulation of both gene sets by the circadian clock. Moreover, we also uncovered distinct overrepresented functions in the induced and repressed gene sets, in accord with previous studies in other examined PIF-regulated processes. Interestingly, promoter analyses showed that, whereas PIF/SD-induced genes are enriched in direct PIF targets, PIF/SD-repressed genes are mostly indirectly regulated by the PIFs and might be more enriched in ABA-regulated genes.

## Introduction

Environmental light conditions change within each day and season, and plants have adapted to these oscillations in solar energy (and the related changes in temperature and water availability) by timing their physiological responses to specific times of the day and/or the year. Of particular importance in this respect is the diurnal photoperiod (or more accurately, the duration of the daily dark period), primarily sensed by the phytochrome (phy) family of photoreceptors (phyA to phyE in *Arabidopsis*; Rockwell et al., 2006; Galvao and Fankhauser, 2015). An important aspect of phy function resides in the ability to bind in its photoactivated state to the PIF (Phy-Interacting Factor) family of basic helix-loop-helix (bHLH) transcriptional regulators (Ni et al., 1999; Bae and Choi, 2008; Leivar and Monte, 2014; Xu et al., 2015). This phy-PIF interaction induces the rapid degradation of the PIF quartet (PIFq) PIF1, PIF3, PIF4, and PIF5 (Bauer et al., 2004; Al-Sady et al., 2006; Lorrain et al., 2008; Shen et al., 2008; Ni et al., 2013), altering the transcription of the target gene network within minutes (Jiao et al., 2007; Pfeiffer et al., 2014).

After seed germination and emergence into sunlight, *Arabidopsis* seedlings are subjected to alternating light/dark cycles. Hypocotyl elongation under these conditions depends on the duration of the dark period in a non-linear fashion, and it is accelerated specifically in the long nights of short-day (SD) photoperiods (Niwa et al., 2009). In SD, seedlings display diurnal rhythmic growth with maximal growth rates at dawn, which are rapidly reduced during the first hours of light (Nozue et al., 2007). In comparison, growth is greatly reduced under SD-entrained seedlings released into constant light (LL; Soy et al., 2014), which indicates that the dark period is necessary for the induction of elongation. The promotion of growth at the end of the night involves the combined actions of PIF1, PIF3, PIF4, and PIF5 (Nozue et al., 2007; Niwa et al., 2009; Soy et al., 2012, 2014). Precise regulation of their accumulation and time of action under diurnal conditions has been proposed to involve different parallel mechanisms. First, at the transcriptional level, *PIF4* and *PIF5* genes are directly targeted by several clock components that impose an internal rhythm of expression with increased transcript levels at the end of the night (Nozue et al., 2007; Nusinow et al., 2011; Huang et al., 2012). In contrast, *PIF1* and *PIF3* transcript levels are maintained fairly constant during the diurnal cycle (Soy et al., 2012, 2014). Second, at the post-transcriptional level, phy-induced degradation imposes oscillation of PIF3 and likely PIF1 proteins to peak at dawn (Soy et al., 2012, 2014), and fine-tunes the timing of PIF4 and PIF5 accumulation (Nozue et al., 2007; Yamashino et al., 2013). In addition, PIF4 has been proposed to be a target of the kinase BIN2 to mark it for proteasome regulation (Bernardo-García et al., 2014). Third, DELLAs and ELF3 have been shown to interfere with DNA binding of the PIFs (de Lucas et al., 2008; Feng et al., 2008; Arana et al., 2011; Nieto et al., 2015). Finally, we have recently shown that the transcriptional activity of PIF3 (and possibly of the other PIFq members) is inhibited by the core component of the circadian clock TOC1 to gate hypocotyl elongation to the end of the night (Soy et al., 2016).

Previous work has established that PIFq stimulation of hypocotyl elongation under SD involves the direct regulation of growth-related genes such as *PIL1 (PHYTOCHROME-INTERACTING FACTOR-3 LIKE 1)*, *HFR1 (LONG HYPOCOTYL IN FAR-RED 1), XTR7 (XYLOGLUCAN ENDOTRANSGLYCOSYLASE 7), GA2OX6*, and *PAR1* (Soy et al., 2012, 2016), which are up-regulated in conditions where hypocotyl elongation is induced (Salter et al., 2003; Lorrain et al., 2008; Hornitschek et al., 2009; Leivar et al., 2009; Nozue et al., 2011), and the regulation of auxin-related genes that oscillate in phase with hypocotyl growth (Michael et al., 2008a; Nozue et al., 2011). Growth-related genes regulated by PIF4 and PIF5 under photoperiodic conditions were defined by Nozue et al. (2011). Many of these genes are direct targets of both PIF and TOC1 and their expression is limited to the pre-dawn hours (Soy et al., 2016). However, a comprehensive analysis of the PIFq-regulated transcriptome under SD conditions is still lacking.

Here, using microarray analysis, we identify genes specifically regulated at the end of the night during the growth conditions of SD compared to LL, where growth is marginal, and define their dependence on PIFq for regulation. Our analysis provides a comprehensive description of the SD- and PIFq-regulated transcriptome at dawn, which identifies ∼60% and ∼40% of genes that are PIF-induced and –repressed, respectively. Additionally, we show that the pattern of rhythmic expression of the PIF/SD-gene set and the contribution of PIFq is markedly different in PIF/SD-induced and PIF/SD-repressed genes. Finally, we describe a functional and promoter sequence dichotomy between the PIF/SD-induced and -repressed gene sets.

## Materials and Methods

### Plant Materials and Growth Conditions

**FIGURE 1:**
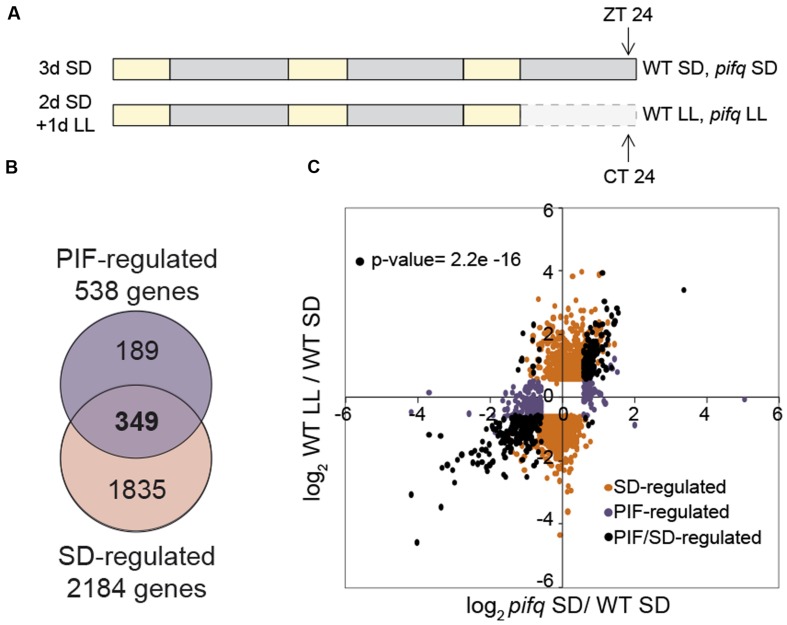
**Overlap between SD and PIF-regulated genes at dawn defines 349 PIF/SD-regulated genes.** **(A)** Schematic representation of seedling growth used for microarray-based transcriptome analysis. Samples of wild type (WT) and *pifq* seedlings grown for 3 days under short days (3dSD), or 2 days in SD plus 1 day in free running conditions (2dSD + 1dLL) were harvested at the end of the night at ZT24 or CT24, respectively. **(B)** Venn diagram shows pairwise comparison between SS1.5F differentially expressed genes comparing WT SD and *pifq* SD (PIF-regulated genes), and WT SD and WT LL (SD-regulated genes). The number of genes that are differentially expressed in each set is indicated. The list of genes in each class is provided in **Dataset S1**. SS1.5F: genes whose expression changed statistically significantly and by at least 1.5 fold. **(C)** Scatterplot of log_2_ fold change (FC) values shows for each gene a quantitative measure of the correlation in responsiveness between the PIF- and SD-regulation described in B: Black dots represent genes that are shared between the two combinations, whereas orange (SD-regulated) and purple (PIF-regulated) dots represent genes that are specifically present in one of the combinations but not in the other (*p*-value = 2.2e-16, one-sided binomial test between quadrants).

Three biological replicates of *Arabidopsis thaliana* wild type (WT) Col-0 and *pifq* mutant seeds (Leivar et al., 2008) were plated on GM medium without sucrose at room temperature as described (Monte et al., 2003). Seedlings were then stratified for 4 days at 4°C in darkness, and then placed in SD conditions [8 h white light (85 μmol m^-2^ s^-1^) + 16 h dark] at the beginning of the light period, considered as day 1. On day 3, seedlings were either kept under SD or transferred to continuous light (LL; Soy et al., 2014). Samples were harvested at the end of the dark period (ZT24, SD samples) or the subjective night (CT24, LL samples; **Figure 1A**).

### Microarray-Based Expression Profiling: Sample Preparation and Data Analysis

Total RNA was prepared using the RNeasy Plant Mini Kit (Qiagen). cRNA synthesis, and microarray hybridizations and washes were performed as described by Affymetrix in the Genomics Facility at CRAG. *Arabidopsis* Affymetrix Gene 1.0 ST arrays were used for gene expression detection.

Data analysis was performed using the Rosetta Resolver Gene Expression Analysis System, version 7.0 (Rosetta Biosoftware) as previously described (Sentandreu et al., 2011). Briefly, two separate statistical analyses were carried out to define (a) a “SD-regulated” gene list of transcripts whose expression is significantly altered by in the WT upon exposure to long night (SD) compared to subjective night (LL); and (b) a “PIFq-regulated” gene list of transcripts whose expression is significantly altered in *pifq* compared to WT under SD. These gene lists were calculated by performing a two-group, two-way, error-weighted, Benjamini-Hochberg false discovery rate error-corrected analysis of variance with a *p*-value cutoff of 0.05. This statistical significance test was combined with experimental consistency by further reducing the statistically significantly (SS) transcript list to only those transcripts exhibiting an absolute FC of greater than 1.5-fold (SS1.5F genes). PIF/SD-regulated genes were defined as SS1.5F genes in both SD- and PIFq-regulated gene lists.

### Promoter Analysis for DNA Binding Motifs

Promoter analysis was performed within 3 kb upstream of the translation initiation site using the Patmach and Motif analysis tools available at the TAIR website^1^. For enrichment analysis performed for each of the investigated motifs, the total number of genes on the *Arabidopsis* Affymetrix Gene 1.0 ST array containing at least one motif of interest, was compared with the total number of genes within our PIF/SD-regulated subset of genes that contain the motif. SS (*p*-value ≤ 0.05) enrichment of these motifs in the various gene subsets was then calculated from the hypergeometric distribution. Promoter analysis using the SCOPE motif finder^2^ was performed within 2.5 kb upstream of the translation initiation site.

### Statistics of Gene Expression Analysis

**FIGURE 2:**
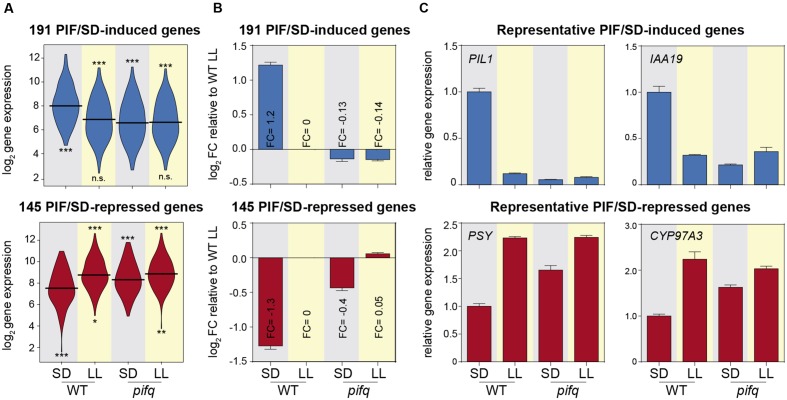
**Contribution of PIFq to the expression of PIF/SD-regulated genes.** **(A)** Vioplot of the log_2_ expression values of the 191 PIF/SD-induced (top) and the 145 PIF/SD-repressed genes (bottom) in the WT and *pifq* SD and LL microarray samples. Statistically significantly (SS) differences from WT SD or *pifq* SD by Willcoxon test are indicated in the upper and lower part, respectively (^∗^*p*-value < 0.05; ^∗∗^*p*-value < 0.01; ^∗∗∗^*p*-value < 0.001. n.s., non-significant). **(B)** Bar graph shows the mean ± SE of log_2_ FC values for PIF/SD-induced (top) and repressed (bottom) genes relative to the expression value of WT LL sample. The mean FC value for each sample is indicated. **(C)** Bar graph of microarray data showing the FC in gene expression relative to the WT SD. Relative expression of two representative PIF/SD-induced genes (*PIL1* and *IAA19*; top) and repressed genes (*PSY* and *CYP97A3*; bottom). Data correspond to biological triplicates, and bars indicate SE.

Binomial test was performed in **Figure 1C** to asses the association between the PIFq and SD regulation of PIF/SD-regulated genes. Differential gene expression shown in **Figure 2A** and **Supplementary Figures S2** and **S6** was determined by Willcoxon test. SS differences are indicated (^∗^*p*-value < 0.05; ^∗∗^*p*-value < 0.01; ^∗∗∗^*p*-value < 0.001).

### Functional Categorization

PIF/SD-regulated genes were functionally categorized according to their established or predicted subcellular localization. A single subcellular localization was assigned to each locus and designations were determined using the annotation of the *Arabidopsis* genome (TAIR10). To identify enriched gene ontology (GO) biological processes, analyses were performed using the functional annotation classification system DAVID^3^ (Huang et al., 2007).

### Data Analyses

Phases of expression were analyzed using the publicly available gene phase analysis tool PHASER^4^ (Mockler et al., 2007) using a cutoff of 0.7. Transcript abundance of diurnal and circadian photocycles was analyzed using the publicly available genome-wide expression data in DIURNAL^5^ (Mockler et al., 2007) for the following conditions: SD (Col-0_SD), 12:12 (Col_LDHH), and Free Running (LL23_LDHH).

## Results

### Night-Induced Transcriptomic Changes under SD Underlying PIFq-Regulated Growth

To delineate the transcriptomic changes underlying PIFq induction of growth under short days (SDs), we performed microarray analysis to define gene expression in 3 day-old WT and *pifq* seedlings grown under SD for 2 days and then kept in SD (3dSD) or released into continuous white light (LL) for an additional day (2dSD+1dLL; **Figure 1A**). This experimental configuration was designed based on our previous results showing that the hypocotyl of seedlings in SD elongate during the night period, whereas seedlings transferred to LL do not grow during the subjective night (Soy et al., 2014). Thus, SD and LL correspond to “night-induced growth” and “no night-induced growth” conditions, respectively, correlating with presence (SD) or absence (LL) of PIF accumulation (Soy et al., 2012, 2014). Samples were collected at dawn in SD (ZT24) when PIF abundance is greatest (Soy et al., 2012, 2014; Yamashino et al., 2013) and at subjective dawn in LL (CT24; **Figure 1A**).

Microarray analysis shows that the expression of 2,184 genes changes statistically significantly (SS) and by 1.5 fold (SS1.5F) during growth in SD relative to LL (WT-SD vs. WT-LL; **Figure 1B** and **Supplementary Figure S1**; see **Dataset S1** online for the gene list). By comparison, *pifq* seedlings in SD display SS1.5F alterations in the expression of 538 genes relative to the SD-grown wild type (WT-SD vs. pifq-SD; **Figure 1B** and **Supplementary Figure S1**; see **Dataset S1** online for the gene list). Of these 538 genes, 349 (64.8%) are identical to those altered in expression in the WT by exposure to the long night of SD (**Figure 1B** and **Supplementary Figure S1**). Of these common 349 SS1.5F genes, 191 (54.7%) are SD- and PIF-induced (i.e., are up-regulated in the WT in SD compared to LL, and down-regulated in *pifq* compared to the WT in SD), and 145 (41.5%) are SD- and PIF-repressed (i.e., are down-regulated in the WT in SD compared to LL, and up-regulated in *pifq* compared to the WT in SD; ??), and were designated PIF/SD-induced and PIF/SD-repressed, respectively (**Dataset S1**). The remaining 13 genes (0.03%) displayed opposite direction of their response between night and PIFq, and were designated as ambiguous (??, **Dataset S1**). The scatterplot shown in **Figure 1C** illustrates the significant strong qualitative and quantitative correlation between the expression elicited by the long night and by PIFq in SD compared with LL for the 349 regulated genes (black symbols; *p*-value = 2.2e-16). In addition, this plot reveals that genes that are SD-regulated only (orange symbols) responded similarly or with only slight differences in expression in the *pifq* compared to the WT (**Figure 1C** and **Supplementary Figure S2A**), indicating that they are only marginally regulated by the PIFs. However, a considerable additional number of the SS1.5F genes that respond to SD in the *pifq* mutant only (PIF-regulated only, purple symbols) also respond in the WT in SD compared to LL, albeit to a lesser extent compared to the fold change (FC) difference between WT and *pifq* (**Figure 1C** and **Supplementary Figure S2B**). Together, these data indicate that there are extensive similarities in the direction and extent of the gene expression changes induced by genetic removal of the PIFq proteins in SD and the changes elicited by suppression of the night period in SD-entrained seedlings released in continuous light, and suggest that the reciprocal regulation by light and PIFs previously described to take place during deetiolation (Leivar et al., 2009) is also reproduced under SD conditions. The degree of this reciprocal regulation was most significant for the identified PIF/SD-regulated subset of 349 genes (**Dataset S1**). These PIF/SD-regulated genes were therefore considered as good candidates to underlie the PIFq-regulated changes in seedling growth and development under SD conditions, and were selected for further analysis.

### PIFq Contribution to the Expression of PIF/SD-Regulated Genes

To estimate the contribution of the PIFq proteins to the night-induced expression of the PIF/SD-regulated genes, we calculated the average contribution of PIFq to the night-induced changes in expression elicited in the WT in SD compared to LL. The expression levels of the 191 PIF/SD-induced gene-set in *pifq* seedlings grown in SD resembled those in WT in LL (and also in *pifq* in LL), indicating that PIFq proteins are necessary to induce their gene expression during the night period under SD (**Figure 2A**). Examination of the contribution of PIFq to the expression of the PIF/SD-induced genes shows that, compared to the normalized log_2_ expression of genes in WT LL conditions set at 0, the mean log_2_ of the FC of WT SD is 1.2, whereas in *pifq* SD and LL is -0.13 and -0.14, respectively (**Figure 2B**). Similarly, for the 145 PIF/SD-repressed gene set, expression levels in *pifq* seedlings grown in SD were more similar to those in LL-grown WT (and also *pifq*), than to SD-grown WT, although there were significant differences among them (**Figure 2A**). In accord, the mean log_2_ FC of WT SD was (-1.3) compared to the WT LL, whereas the mean FC in *pifq* SD was (-0.4; **Figure 2B**). The mean log_2_ of the FC expression in *pifq* LL was 0.05, similar to WT LL (**Figure 2B**). When these differences between WT and *pifq* are expressed as percentage contribution of PIFq to the change induced in the WT in SD compared to LL, these results indicate that, on average, PIFq contributes to 100% of the upregulation of PIF/SD-induced genes, whereas their contribution to the repression of gene expression in WT SD compared to WT LL is ∼69%, suggesting that other factors must be responsible for the remaining 30% change in expression. To visualize the array of the contribution of PIFq to the expression of the individual genes under SD, we ranked the percent-contribution values for the induced and repressed genes separately (**Supplementary Figure S3**). All of the PIF/SD-induced genes showed a contribution larger than 50%, with 64% of the genes having a contribution that was 100% or greater. In contrast, of the PIF/SD-repressed gene set, 35% of the genes contributed less than 50%, and only 14% of the genes had a contribution that was 100% or higher (**Supplementary Figure S3**). Together, the data suggest that gene induction and repression under SD might be differently regulated by the PIFs: whereas the PIFq appears to be necessary and sufficient to explain the night induction of PIF/SD-induced genes, other proteins might be involved in the night repression of PIF/SD-repressed genes. As examples of the contribution of PIFq for specific PIF/SD-regulated genes, **Figure 2C** shows the expression of representative PIF/SD-induced genes *PIL1* (*AT2G46970*) and *IAA19* (*AT3G15540*), and PIF/SD-repressed genes *PSY* (*AT5G17230*) and *CYP97A3 (AT1G31800)*, in WT and *pifq* seedlings grown under SD or LL.

### Phase Enrichment and Diurnal Expression of PIF/SD-Regulated Genes

**FIGURE 3:**
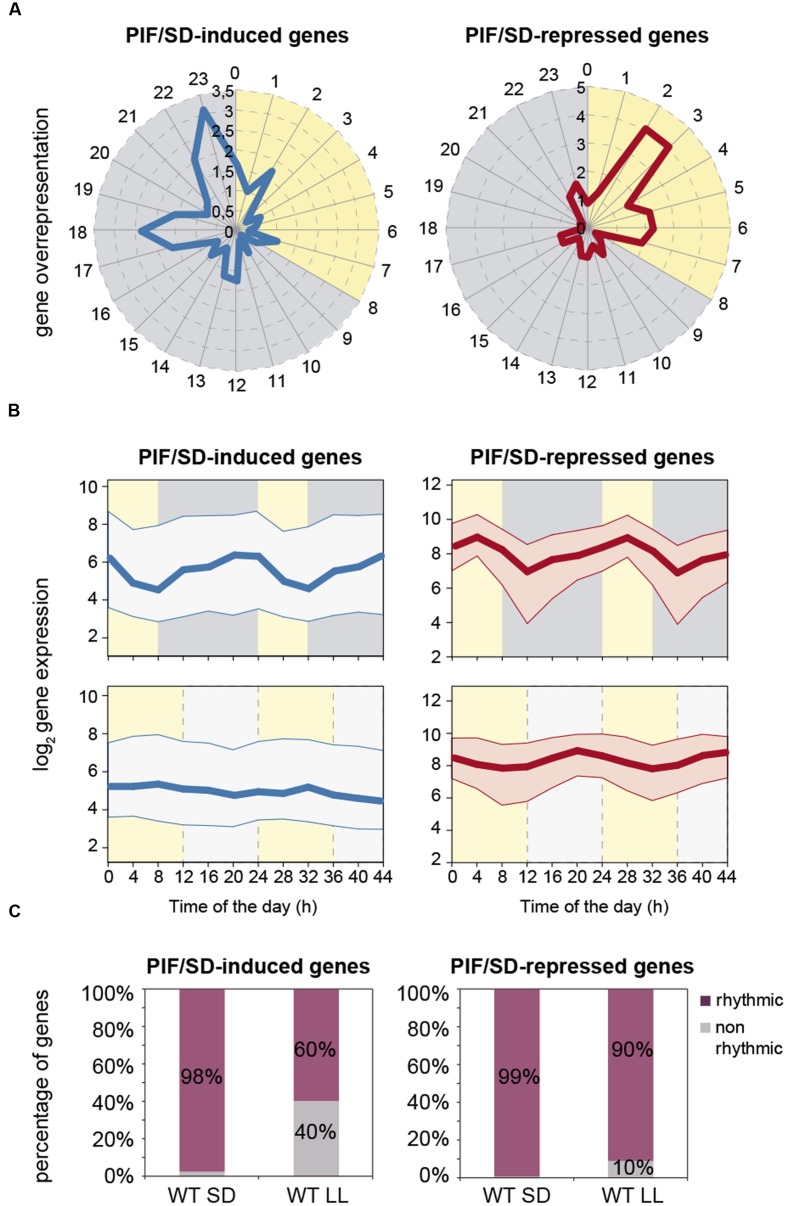
**Expression dynamics of PIF/SD-induced and -repressed genes under SD compared to LL photoperiods.** **(A)** Expression phases in SD of the 191 PIF/SD-induced (left) and the 145 PIF/SD-repressed (right) genes. Phases, defined by PHASER (http://phaser.mocklerlab.org), are indicated on the circumference, and FC phase enrichment of genes (count/expected) on the radius. Day: yellow; Night: gray. **(B)** Median values (thick line) and upper and lower quartiles (thin lines) of log_2_ expression for all PIF/SD-induced (left) and PIF/SD-repressed (right) under SD (top) compared to free running conditions in seedlings entrained in 12:12 (bottom). See **Supplementary Figure S4** for values in 12:12. Data were obtained from http://diurnal.mocklerlab.org. Day: yellow; Night (top): gray; Subjective night (bottom): light gray **(C)** Percentage of rhythmic genes in SD or LL in the PIF/SD-induced (left) and the PIF/SD-repressed (right) gene sets. Data are from http://phaser.mocklerlab.org.

Time-of-day-expression enrichment-analysis of the PIF/SD-regulated genes was performed using the available data at the PHASER website^6^ (Mockler et al., 2007). The data showed that, under SD photocycles, PIF/SD-induced genes displayed an overrepresented phase of expression at the end of the dark period (**Figure 3A**), with 49% of these genes phased at pre-dawn between 18 and 23 h (**Dataset S1**), when PIF abundance is maximum. By contrast, the phase-overrepresentation pattern was remarkably different for the PIF/SD-repressed genes, which displayed an overrepresented phase of expression in the morning (**Figure 3A**), with 26% of these genes phased between 2 and 3 h (**Dataset S1**). Consistent with this phase enrichment, the expression of PIF/SD-regulated genes under SD shows an oscillatory pattern (**Figure 3B**, top). The expression of PIF/SD-induced gene set is kept low during the day, progressively increases during the night and displays a maximum at the end of the dark period (**Figure 3B**, top). In contrast, the PIF/SD-repressed gene set presents a similar increase during the night but the peak of maximum expression occurs during the day (**Figure 3B**, top). Interestingly, these oscillatory patterns are not maintained under free-running conditions (**Figure 3B**, bottom). Indeed, expression for PIF/SD-induced genes is kept at low levels across the day and the subjective night under LL (**Figure 3B**, bottom left). Strikingly, for PIF/SD-repressed genes, whereas, the expression in LL displays a comparable night increase compared to SD, there is a significant phase shift from mid morning to the end of night (**Figure 3B**, bottom right), also apparent when compared to the corresponding entrainment conditions in 12:12 that also display a mid-morning peak (**Supplementary Figure S4**). Accordingly, whereas most PIF/SD-regulated genes are rhythmic under SD conditions, the percentage of rhythmic genes under free running is reduced to 60% in the PIF/SD-induced gene set, and to 90% in the PIF/SD-repressed gene set (**Figure 3C**). Together, these data suggest that PIF accumulation during the night underlies the phase and amplitude of the rhythmicity of PIF/SD-induced and -repressed genes, to peak at pre-dawn and in the morning, respectively.

### Comparison of PIFq-Regulated Genes in SD with PIF-Regulated Genes During Deetiolation, Shade, or Photoperiodic Growth

**FIGURE 4:**
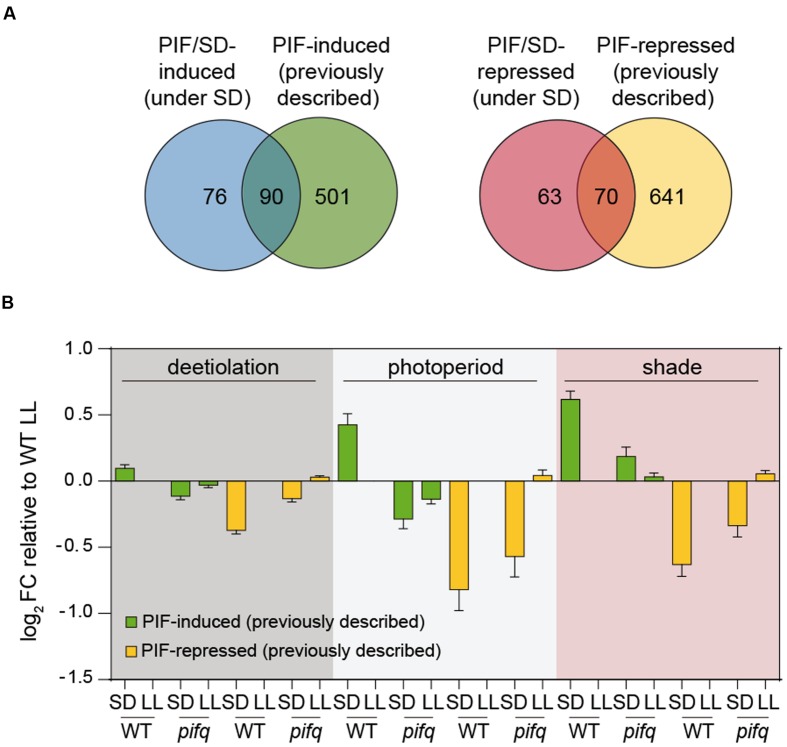
**Comparison of PIF-regulated gene expression in SD with the expression of previously defined PIF-regulated gene sets during deetiolation, shade, and photoperiodic conditions.** **(A)** Venn diagram shows the overlap between the PIF/SD-induced (left) or PIF/SD-repressed (right) genes, and the composite of 591 PIF-induced and 711 PIF-repressed genes previously described during deetiolation (Leivar et al., 2009), photoperiod (Nozue et al., 2011), and shade (Leivar et al., 2012). **(B)** Bar graph of WT and *pifq* microarray data (SD and LL) showing the log_2_ FC expression relative to the WT LL of the PIF-induced (green) or PIF-repressed (yellow) genes defined during deetiolation (left), photoperiod (middle) and shade (right), that are not PIF/SD-regulated in our experimental conditions.

Previously, we defined a common set of PIF-regulated “central-class” genes that were associated to the light/PIF-regulated responses of deetiolation, shade, and diurnal conditions (Leivar and Monte, 2014). These genes might represent common effectors in different light environments to implement PIF-regulated seedling growth and development. To establish whether our PIF/SD-regulated set of genes might also be in common with genes associated to other PIF-regulated developmental processes, we decided to examine how this gene set compares to the previously defined PIF-regulated gene sets. To this end, we first compiled a list of PIF-induced (591) and PIF-repressed (711) genes defined in at least one of previously described conditions where PIFs are regulating development: shade (Leivar et al., 2012), deetiolation (Leivar et al., 2009), and photoperiod (Nozue et al., 2011). Next, we compared it to the subset of PIF/SD-regulated genes described here filtered for presence in the ATH1 microarray platform used in the three abovementioned genomic analyses (166 PIF/SD-induced and 133 PIF/SD-repressed genes). We found that the percentage of overlap was similar for PIF/SD-induced and PIF/SD-repressed, with 54 and 53% of the genes having been previously described as PIF-induced or PIF-repressed, respectively (**Figure 4A**). Of the 22 PIF-induced “central class” genes (Leivar and Monte, 2014), 16 were PIF-induced in all experiments including SD (**Dataset S1**). *AT3G15850*, the only “central class” PIF-repressed gene (Leivar and Monte, 2014), was also a PIF/SD-repressed gene.

To further investigate the light and PIF-mediated regulation of genes not defined as common in all classes, we next examined the behavior of the subsets of genes that (1) were previously defined as PIF-regulated genes but were not in common with our defined PIF/SD-gene set under the diurnal SD conditions assayed here (**Figure 4A**), and (2) were defined here as PIF/SD-genes but were not previously identified as PIF-regulated genes (**Supplementary Figure S5**). For the previously defined PIF-regulated genes not in common with our defined PIF/SD-gene set (501 PIF-induced and 641 PIF-repressed genes; **Figure 4A**), the data show that the average FC expression of these genes followed a pattern similar to the PIF/SD-regulated genes (**Figure 4B**, compare with **Figure 2B**). Indeed, the PIF-induced gene sets in shade (117 genes; Leivar et al., 2012), photoperiod conditions (43 genes; Nozue et al., 2011), and during deetiolation (388 genes; Leivar et al., 2009) that were not in our PIF/SD-regulated gene set, exhibited induced expression under SD compared to WT LL, an induction that was either absent (deetiolation and photoperiod) or much reduced (shade) in *pifq* SD (**Figure 4B**). Similarly, the PIF-repressed gene sets in shade (82 genes; Leivar et al., 2012), in photoperiod conditions (32 genes; Nozue et al., 2011), and during deetiolation (567 genes; Leivar et al., 2009) that were not in our PIF/SD-regulated gene set, exhibited repressed expression under SD compared to WT LL, a repression that was partially reduced in *pifq* SD (**Figure 4B**). These data indicate that previously defined PIF-regulated genes that are not in our PIF/SD-regulated gene set were not selected here because they did not meet the SS1.5 FC requirement in the comparisons of WT SD vs. WT LL and/or *pifq* SD vs. WT SD (**Figure 4B**). However, these PIF-regulated genes follow a similar regulatory pattern under SD compared to the selected PIF/SD-regulated gene set (**Figure 2B**), indicating that they are also regulated in the long nights of SD by the PIFs, albeit to a lesser degree. Notably, PIF-regulated genes during deetiolation have the lowest average FC (WT SD compared to WT LL) under SD conditions (**Figure 4B**). This result suggests that the response of dark-grown seedlings to light might be stronger compared to the response of SD-grown seedlings to the long nights of SD compared to LL, or the response of light-grown seedlings to shade. For the second set of genes that was selected here as PIF/SD-regulated but had not been described before as PIF-regulated during deetiolation, shade or photoperiod (76 PIF-induced and 63 PIF-repressed genes; **Figure 4A**), we asked whether it might represent genes with a specific role under SD conditions, or whether they are genes that were also regulated in the previous deetiolation, photoperiod, or shade studies, but did not meet the FC or SS requirements applied. **Supplementary Figure S5** shows that expression of both induced and repressed PIF/SD-regulated genes followed a similar PIF-dependent expression pattern during deetiolation, photoperiod or shade, albeit with a FC that was reduced compared to SD (**Figure 2B**). We conclude that, to a great extent, the genetic network imposed by the PIFs under different conditions involves the same genes. However, differences in the expression FC comparing dark or shade with light, and/or the contribution of each gene to the overall morphological, cellular, and subcellular phenotype might be specific under each condition.

### Functional Categorization of PIF/SD-Regulated Genes

**FIGURE 5:**
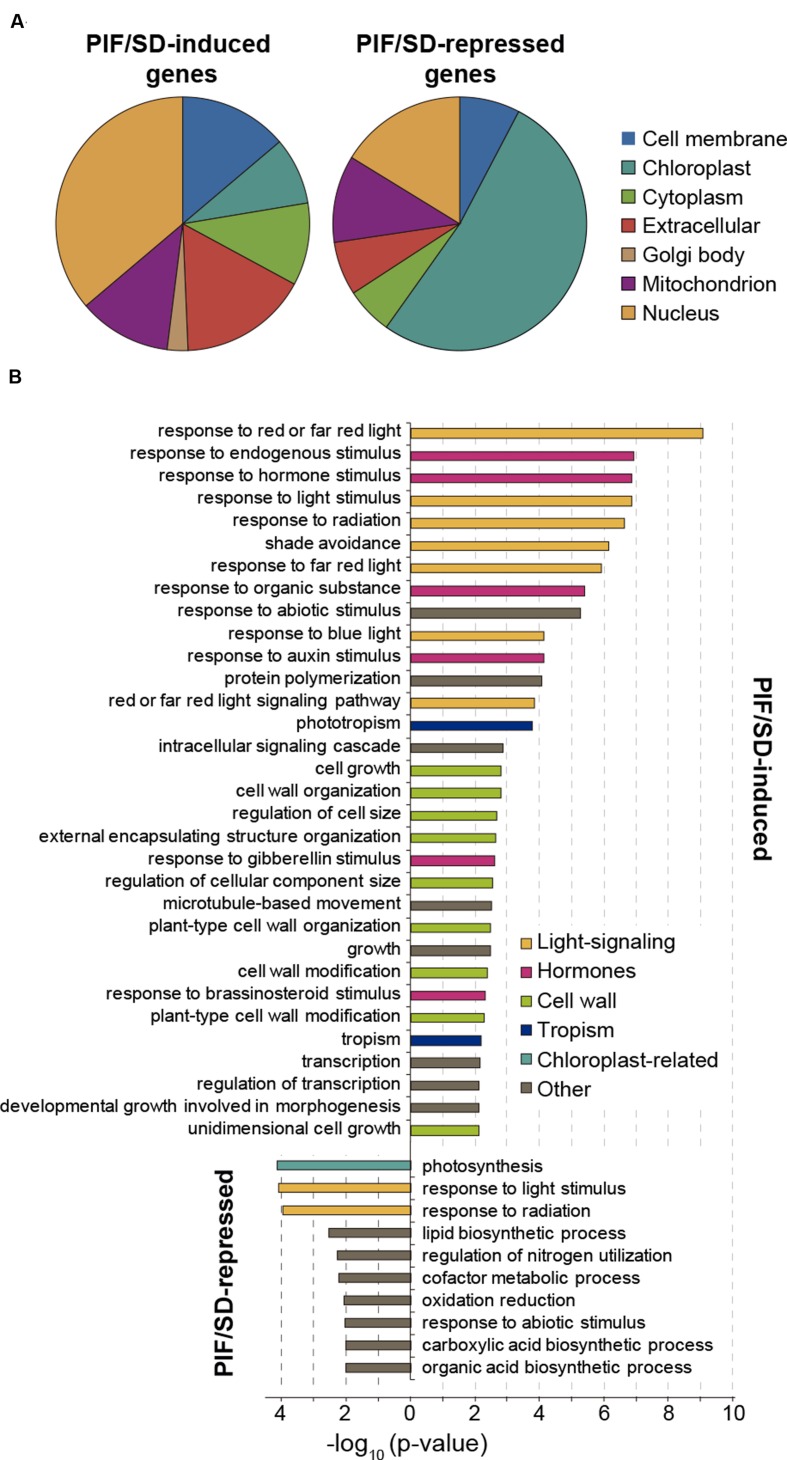
**Subcellular localization and functional analysis of the PIF/SD-regulated genes.** **(A)** Subcellular localization of PIF/SD-regulated genes based on the gene ontology (GO) annotations available at TAIR (http://www.Arabidopsis.org). Each class is represented as percentage of the total annotated genes excluding the genes annotated as having unknown localization. The list of genes belonging to each class is provided in **Dataset S1**. **(B)** Enriched GO categories (biological process) of PIF/SD-induced (top) and -repressed (bottom) gene sets. DAVID *p*-value indicates significance (Fisher’s exact test; *p*-value < 0.05).

The PIF/SD-regulated genes were classified according to their established or predicted subcellular localization and GO terms (**Figure 5**; **Dataset S1**). Of the PIF/SD-induced genes with an assigned subcellular localization, the data show that genes coding proteins that localize to the nucleus are the most abundant (36%; **Figure 5A**). This result is similar to the previously reported abundance of transcription factors among PIF-regulated and PIF-target genes during deetiolation and shade (Monte et al., 2004; Leivar et al., 2009; Hornitschek et al., 2012; Zhang et al., 2013; Pfeiffer et al., 2014) and it is consistent with the PIFs acting as master regulators of a transcriptional network in the adaptation of seedling morphology to alternating day and night conditions. Genes in this category include the transcriptional regulators *PIL1, FHL*, *HFR1*, and *HAT2* (described to be PIF-induced specifically at the end of the night under SD; Nomoto et al., 2012; Soy et al., 2016). Other well-represented subcellular categories are cell membrane (14%) and extracellular (16%) related genes (**Figure 5A**). Among the overrepresented biological processes, PIF/SD-induced genes are significantly enriched in genes involved in responses to light and hormone stimuli, transcription, and cell growth processes (**Figure 5B**). Among the response to hormone stimulus, auxin-related genes are dominant (namely we found four SAUR genes and the IAA genes *IAA2*, *IAA19*, and *IAA29*) and thus “response to auxin stimulus” is strongly enriched (**Figure 5B**). There is also enrichment in the response to gibberellins (GAs) and brassinosteroid (BR) stimuli. The cell growth category includes genes that are involved in cell wall loosening and modification such as the *XYLOGLUCAN ENDOTRANSGLUCOSYLASES* (*XTH*) *XTH8*, *XTH15*, *XTH30*, and *XTH33*, the *CELLULOSE-SYNTHASE-LIKE C4* (*CSLC04*), the *BARWIN-LIKE ENDOGLUCANASES SUPERFAMILY PROTEIN EXP3*, the *EXPANSIN11* (*EXPA11*), and the *EXPANSIN-LIKE A2* (*EXLA2*). This analysis, together with previous studies (Michael et al., 2008a; Leivar et al., 2009; Nozue et al., 2011; Hornitschek et al., 2012; Pfeiffer et al., 2014) suggests that PIFq might induce elongation during the long nights of SD by upregulating the expression of hormonal pathways such as auxin, GA and BR, and genes involved in cell wall modification to support cell growth. This PIFq-mediated induction of growth-related gene expression is suppressed in the morning upon light exposure and consequent reduction in PIF protein levels (**Figure 3**; Nozue et al., 2007; Soy et al., 2012, 2014). Similarly, under LL, PIFs do not accumulate (Soy et al., 2014) and this correlates with reduced expression of growth-related genes and growth arrest under the subjective night of LL compared to the long night of SD (**Figure 3**).

Of the PIF/SD-repressed genes, the data show that genes coding for proteins predicted or established to localize to the chloroplast are the most abundant (52%), followed by the genes assigned to code for proteins localized to the nucleus (16%; **Figure 5A**). Other prominent categories are the mitochondrion (11%) and extracellular (7%; **Figure 5A**). Among the overrepresented biological processes, PIF/SD-repressed genes are significantly enriched in genes involved in photosynthesis and in response to light and radiation stimuli (**Figure 5B**). The photosynthesis/chloroplast categories include genes involved in photosynthesis (e.g., the photosystem I *PGR5-LIKE* A, and the photosystem II *PsbP* and *PSB28)*, genes coding for structural components of the chloroplast [e.g., acyl carrier protein 4 (*ACP4*)], genes necessary for chlorophyll biosynthesis (*CHLI1, HEMA1*), and genes involve and carbon fixation, such as the small chain of the ribulose bisphosphate carboxylase. Interestingly, it also includes the master regulators of chloroplast development *GLK2* and *SIGMA5* (Fitter et al., 2002; Waters et al., 2009; Noordally et al., 2013). It is worth noting that the expression of these genes, even though they are PIF-repressed genes in SD conditions compared to LL, is actually progressively induced during the long night of SD as shown above (**Figure 3B**, top). Together, these results suggest that PIFq prevents over-expression of chloroplast-related genes during the night. Once the seedling is exposed to light, PIFs are degraded, the repression is lifted and these genes reach maximum expression during the morning hours (**Figure 3B**, top).

### Promoter Analysis of PIF/SD-Regulated Genes

**FIGURE 6:**
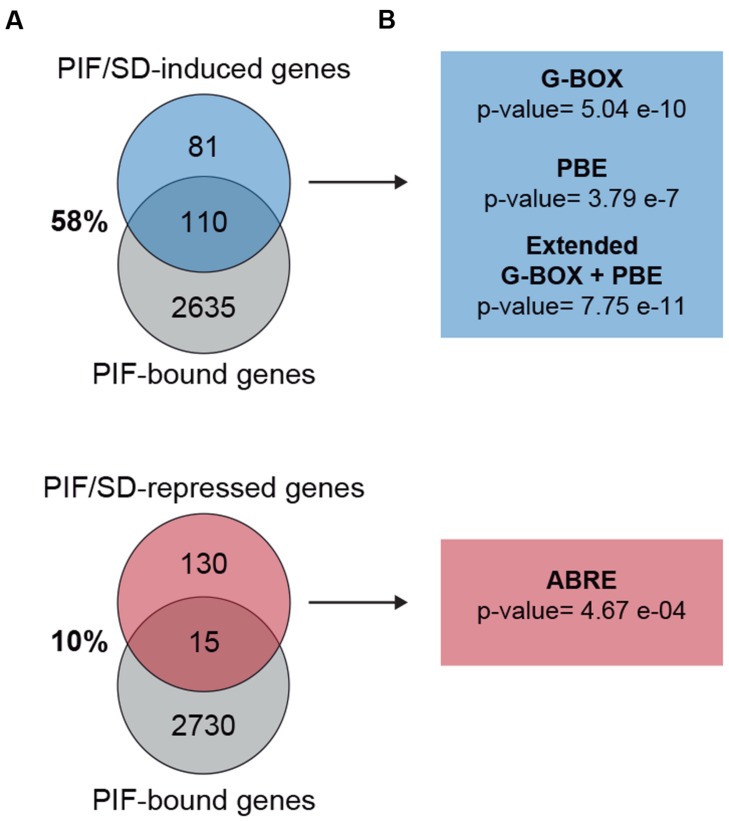
**Promoter analysis of PIF/SD-regulated genes.** **(A)** Venn diagram shows the overlap between the PIF/SD-induced (top) or PIF/SD-repressed (bottom) genes and the previously defined PIF-bound genes (Hornitschek et al., 2012; Pfeiffer et al., 2014). **(B)** Enriched DNA elements in the promoters of PIF/SD-induced (top) or PIF/SD-repressed (bottom) genes.

PIF proteins have been experimentally shown to bind sequence specifically to the G-box motif CACGTG and the PIF-binding element (PBE)/Hormone-up at dawn box (HUD) CACATG (Hornitschek et al., 2012; Oh et al., 2012; Zhang et al., 2013; Pfeiffer et al., 2014). We therefore examined the PIF/SD-regulated genes identified in our analysis for the presence of these motifs within 3 kb upstream of the transcription start site. This analysis is presented in **Figure 6A** and **Table 1**. We found that genes in the induced set displayed SS enrichment of both elements. The G-box and PBE were present in 54% (*p*-value: 5.04E–10) and 90% (*p*-value: 3.79E–07) of the genes, respectively. Similar results were obtained using SCOPE (**Table 1**). These results suggest that a high % of the PIF/SD-regulated genes might be direct targets of the PIFs. Indeed, comparison with previously defined PIF-bound genes in deetiolation and shade (Hornitschek et al., 2012; Pfeiffer et al., 2014) showed that 57.5% of the PIF/SD-induced genes defined here were identified as PIF-bound under these conditions (110 genes; **Figure 6A**). Based on our previous data showing that PIF-bound genes in etiolated and shade-grown seedlings can also be bound by the PIFs under SD (Soy et al., 2012, 2016), we propose that these SD/PIF- induced genes that are PIF-bound are likely direct targets of the PIFs under diurnal conditions.

**Table 1:** DNA motifs associated with PIF/SD-induced and PIF/SD-repressed genes.

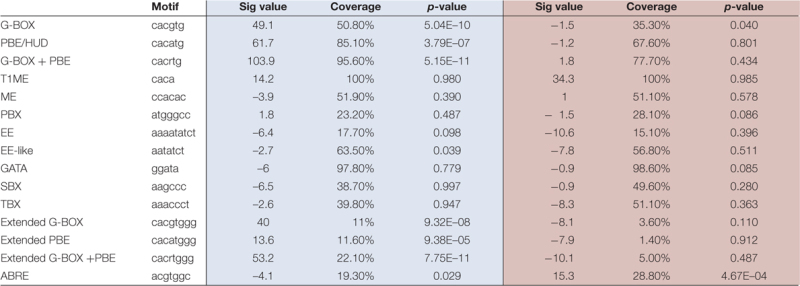

*Presence of previously described diurnal and circadian DNA motifs (Michael et al., 2008b; Soy et al., 2016) in the promoters of PIF/SD-induced (left, in blue) and PIF/SD-repressed genes (right, in red). Analyses were performed using the SCOPE motif finder within 2.5 kb upstream of the translation initiation site (TSS; http://genie.dartmouth.edu/scope/) and the Patmach and Motif analysis tools available at the TAIR website (http://www.Arabidopsis.org/cgi-bin/patmatch/nph-patmatch.pl) within 3 kb upstream of the TSS. Base pair “r” refers to either “g” or “a”. Sig value (a measure of how likely the motif in question is to have been this over-represented. Values greater than zero are meaningful, and the higher the number, the better) and coverage (indicating the percentage of the genes in the query that contain at least one instance of the sequence being examined) are as defined by SCOPE*.

Interestingly, these genes were also enriched (*p*-value: 7.75E–11) in the extended G-box/PBE element (cacrtggg, where r can be a “g” or “a”; **Figure 6A** and **Table 1**) that we have described recently (Soy et al., 2016). This motif was found to be enriched in the flanking regions of the PIF binding sites in genes bound by both PIFs and TOC1 with an expression phase at the end of the night (Soy et al., 2016). This result suggests that this PIF/SD-induced gene set might be enriched in genes that are directly targeted by PIF and TOC1. Out of the 110 PIF-bound genes in the PIF/SD-induced set (Pfeiffer et al., 2014), 11% of the comparable PIF-bound genes (84 genes, as defined in Soy et al., 2016) are predicted to be bound as well by TOC1 (Huang et al., 2012). Conversely, expression analysis of the PIF and TOC1 bound genes that were defined as “predawn-specific PIF-TOC1” (Soy et al., 2016) showed that most are induced at ZT24 compared to LL in the conditions described here, and that this induction is dependent on the presence of PIFq (**Supplementary Figure S6**). As a control, genes defined as PIF and TOC1 bound but not phased at predawn (Soy et al., 2016) do not show this regulation under SD conditions at ZT24 (**Supplementary Figure S6**). Together, these results suggest that a subset of the PIF/SD-induced genes might be directly targeted by PIF and TOC1 under SD, consistent with our recent description of TOC1 repressive action on PIF transcriptional activity as a mechanism to gate growth at dawn (Soy et al., 2016). In agreement, growth-related genes targeted by TOC1 under SD conditions (like *PIL1*, *HFR1*, *CKX5*, *FHL*, or *HAT2*) that were shown to have early expression in a *toc1* mutant under SD conditions (Soy et al., 2016), are included in our PIF/SD-induced gene set (**Dataset S1**).

In contrast to the induced set, we found that genes in the repressed set were only marginally enriched in the G-box and the PBE element, and were not significantly enriched in the combination of G-box+PBE (*p*-value: 0.43; **Figure 6A** and **Table 1**). Accordingly, only 10% of them were previously identified as PIF-bound genes (Hornitschek et al., 2012; Pfeiffer et al., 2014; **Figure 6A**). These results suggest that the majority of the PIF/SD-repressed genes are not direct targets of the PIFs, in agreement with Leivar and Monte (2014) and Martín et al. (2016). Interestingly, analysis of DNA binding motifs revealed that the PIF/SD-repressed gene set is significantly enriched in the ABA-responsive (ABRE) ACGTGGC element (*p*-value: 0.00046, present in 28% of the genes). Similar results were obtained using SCOPE (**Table 1**). This finding suggests that changes in ABA levels or response might underlie the PIF-mediated down-regulation of these genes at the end of the night.

## Discussion

In *Arabidopsis* seedlings grown under SD conditions, PIF activity peaks at dawn coinciding with maximum PIF protein levels and declining abundance of their repressor TOC1 (Soy et al., 2016). The genomic analysis presented here defines the PIFq-regulated transcriptome at the end of the night in SD, and establishes that approximately 60% of the SD- and PIFq-regulated genes correspond to induced genes, whereas 40% are SD- and PIFq-repressed. The data identify a contrasting PIFq contribution to the expression, rhythmicity, and function in the induced and repressed gene sets, and suggest different PIF-regulatory mechanisms to control growth and other aspects of seedling development under SD.

Our observation that PIF-induced genes oscillate in SD but are not rhythmic under LL expands our recent observation for a smaller subset of PIF-induced genes (Soy et al., 2016). This result provides additional supporting evidence that PIF-induced genes at dawn do not cycle in conditions where PIFs do not accumulate (Soy et al., 2014, 2016), and therefore cannot be considered classical clock outputs. Interestingly, our results showing that 11% of the PIF/SD-induced genes are directly targeted by PIF and TOC1 is in accordance with our recent description of the TOC1/PIF antagonistic interplay to gate growth at the end of the night (Soy et al., 2016). Additionally, because both the TOC1 and PIFq ChIP-seq experiments referred to in here were performed under conditions different to SD (12:12 and deetiolation, respectively; Huang et al., 2012; Pfeiffer et al., 2014), the actual % of PIF-TOC1 targets among the PIF/SD-induced genes might be larger. For example, the PIF direct target *PIL1* was not identified as TOC1 target by Huang et al. (2012), whereas we have experimentally shown that it is bound by TOC1 under SD conditions (Soy et al., 2016). Further experiments directed to specifically determine the genes bound by TOC1 and PIFs under SD are needed to establish a comprehensive list of PIF and TOC1 target genes under the SD conditions assayed here.

In contrast to the PIF/SD-induced set, our observation that the repressed gene set is still largely rhythmic under LL (**Figure 3**) suggests that these genes are likely clock-output genes. This establishes a mechanistic difference in how induced and repressed genes are regulated under SD. Interestingly, in addition, the expression dynamics of the repressed gene set is not the mirror image of the induced set, i.e., they are not genes that are progressively repressed during the night as a result of PIF accumulation. Instead, the PIF/SD-repressed genes are also progressively induced during the night (**Figure 3**). The mechanism underlying this pattern is currently not understood, and could involve induction by yet an unknown mechanism. In the WT, these genes peak in the morning, whereas genetic removal of the PIFs induces higher accumulation at dawn. These observations indicate that the PIFs are suppressing an early transcription induction during the last part of the night, and suggest that the PIFs are actively preventing these genes to peak during the night. In the morning, when PIF levels are low due to phytochrome-induced degradation, these genes are induced and display their maximum peak of expression.

Our findings that the promoters of PIF/SD-induced genes are enriched in the G-box and in the PBE/HUD elements, and that the PIF/SD-induced set is enriched in putative PIF-bound genes (**Figure 6**), agree with previous studies (Leivar et al., 2009; Zhang et al., 2013; Leivar and Monte, 2014; Pfeiffer et al., 2014) and are in line with the PIFs acting as transcriptional activators (Huq et al., 2004; Al-Sady et al., 2008; de Lucas et al., 2008; Shen et al., 2008; Hornitschek et al., 2009). The PBE/HUD element was found to be enriched in the promoters of hormone-related genes induced at dawn (Michael et al., 2008a). Accordingly, we found enrichment in growth- and hormone-related genes in the PIF/SD-induced set (**Figure 5**). Interestingly, the induced set was also enriched in the extended G-box/PBE element that we have recently described in the promoters of predawn-specific PIF and TOC1 cotarget genes (Soy et al., 2016), which agrees with the PIF/SD-induced set being co-targeted antagonistically by both factors to time growth.

Importantly, although we found that the PIF/SD-repressed gene set is not enriched in putative PIF-bound genes, some of the repressed genes are also likely targeted directly by the PIFs (10%, 15 genes; **Figure 6**; Pfeiffer et al., 2014), suggesting that the PIFs might also act as transcriptional repressors under SD, in accord with previous results during the deetiolation response (Toledo-Ortiz et al., 2010; Chen et al., 2013; Liu et al., 2013; Zhang et al., 2013; Leivar and Monte, 2014; Pfeiffer et al., 2014). Notably, the master regulators of chloroplast development *SIGMA5* and *GLK2* (Waters et al., 2009; Noordally et al., 2013; Martín et al., 2016) are among the PIF/SD-repressed and PIF-bound genes (Oh et al., 2012; Pfeiffer et al., 2014; Song et al., 2014). This finding suggests that they could act as intermediaries between the PIFs and the chloroplast-related PIF/SD-repressed genes that are not directly bound by the PIFs, delivering the PIF signal to the downstream targets indirectly repressed by PIFq. The mechanism of how PIFs repress gene expression is still largely unknown, but a recent paper showing that PIF3 interacts with the deacetylase HDA15 to repress chlorophyll biosynthesis and photosynthetic genes (Liu et al., 2013) supports the notion that PIFs might associate with repressive histone-modification factors to function as transcriptional repressors. Additionally, our finding that PIFq cannot explain 100% of the repression (in contrast to the PIF/SD-induced genes set, apparently regulated solely by PIFq) agrees with the possibility that part of the repression mechanism might involve chromatin-remodeling events independent of PIFq. Alternatively, factors other than PIFq that accumulate or are activated in the dark could be contributing to the repression of these genes in SD compared to LL. Finally, our finding that the PIF/SD-regulated genes (and more significantly the repressed set) are enriched in genes containing the ABA-responsive element ABRE opens the intriguing possibility that ABA mediates some aspects of PIF/SD-regulated development. To our knowledge, ABRE has not been recognized before as an overrepresented DNA binding element for PIF-regulated genes, which might indicate a role for ABA in PIF-regulated development specifically under SD. The hormone ABA has been described to regulate growth, stomata opening and stress responses (Finkelstein, 2013). Further studies are necessary to explore the possible involvement and role of ABA under the SD conditions examined here.

## Additional Information

Microarray data reported in this study have been deposited in the Gene Expression Omnibus database under the accession number GSE81813.

## Author Contributions

JS and EM designed and performed the research. GM and EM analyzed the data and wrote the manuscript.

## Conflict of Interest Statement

The authors declare that the research was conducted in the absence of any commercial or financial relationships that could be construed as a potential conflict of interest.

## Footnotes

1 http://www.Arabidopsis.org/

2 http://genie.dartmouth.edu/scope/

3 http://david.abcc.ncifcrf.gov

4 http://phaser.mocklerlab.org

5 ftp://diurnal.mocklerlab.org

6 http://phaser.mocklerlab.org

## Supplementary material

**Definition of 191 PIF/SD-induced and 145 PIF/SD-repressed genes. (A)** Venn diagram showing the four-way comparison between SS 1.5F PIF-regulated (WT SD vs. *pifq* SD, induced and repressed) and SD-regulated (WT SD vs. WT LL, induced and repressed) gene sets. The number of differentially expressed genes in each set is indicated. The list of genes in each class is provided in **Dataset S1**. SS1.5F: genes whose expression changed statistically significantly and by at least 1.5 fold. **(B)** Two-dimensional-cluster diagram depicting the identified PIF- regulated SS1.5F genes in 3-day-old SD- and LL-grown WT and *pifq* seedlings. A total of 207 genes are upregulated (PIF-repressed) in the absence of PIFq, whereas 331 correspond to genes that are downregulated (PIF-induced), as defined in **(A)**.

FIGURE S2 **Gene expression of SS1.5F PIF-specific and SD-specific regulated genes. (A)** Vioplots of log_2_ expression values in WT and *pifq* (SD and LL) of the 881 SD-induced (left) and the 954 SD-repressed (right) genes that are not regulated by PIFq, as defined in **Supplementary Figure S1A**. **(B)** Vioplots of log_2_ expression values in WT and *pifq* (SD and LL) of the 130 PIF-induced (left) and the 59 PIF-repressed (right) genes that are not regulated by SD, as defined in **Supplementary Figure S1A**. **(A,B)** Statistically significant differences from WT SD or WT LL by Willcoxon test are indicated in the upper and lower part, respectively (^∗^*p*-value < 0.05; ^∗∗^*p*-value < 0.01; ^∗∗∗^*p*-value < 0.001. n.s., non-significant). The mean log_2_ FC value relative to the expression value of WT LL for each sample is indicated.

FIGURE S3 **PIF/SD-regulated genes ranked based on the percentage contribution of PIFq to their expression in SD**. PIF/SD-induced (left) and PIF/SD-repressed genes (right) were arranged in descending percentage based on the contribution of PIFq to their expression in SD. Percentage of genes with a contribution of <50% or >100% is indicated for each gene set.

FIGURE S4 **Diurnal pattern of PIF/SD-regulated genes in 12:12. Median values (thick line) and upper and lower quartiles (thin lines) of log_2_ expression for all PIF/SD-induced (left) and PIF/SD-repressed (right) under 12:12**. Data were obtained from http://diurnal.mocklerlab.org. Day: yellow; Night: gray.

FIGURE S5 **Expression of the SD-specific PIF/SD-regulated genes under the previously described shade, deetiolation and photoperiodic growth conditions**. Bar graph of microarray data [deetiolation (Leivar et al., 2009), shade (Leivar et al., 2012), and photoperiodic conditions (Nozue et al., 2011)] showing the log_2_ FC expression relative to the WT Light in deetiolation and shade, and relative to *pif4pif5* in growth phase, of the 76 PIF/SD-induced (top) and 63 PIF/SD-repressed (bottom) genes not previously defined as PIF-regulated in any of the other experimental conditions. SS, statistically significantly; FRed: Far red.

FIGURE S6 **Expression of the previously defined “dawn-specific PIF-TOC1” and “no dawn-specific PIF-TOC1” in SD conditions**. Box plots of microarray data showing the log_2_ FC expression in WT and *pifq* in SD and LL, relative to the WT LL, of the “dawn-specific PIF-TOC1” (green) and “no dawn-specific PIF-TOC1” (purple) genes recently defined by Soy et al. (2016). Statistically significant differences among FC values relative to WT SD by Willcoxon test are indicated (^∗^*p*-value < 0.05; ^∗∗∗^*p*-value < 0.001. n.s., non-significant).

DATASET S1 **Expression data and characterization (PIF- and SD-regulation, gene class, phase, and subcellular localization) of the 2,373 SD- and/or PIF-regulated genes identified**.
