# Supplementary figures and images for: Genomic Analysis Reveals Contrasting PIFq Contribution to Diurnal Rhythmic Gene Expression in PIF-Induced and -Repressed Genes
Source: Front Plant Sci. 2016 Jul 4;7:962. doi: 10.3389/fpls.2016.00962 (PMC4930942; doi:10.3389/fpls.2016.00962)

**A**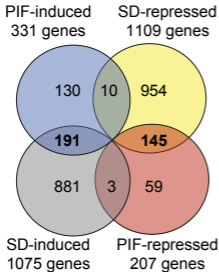**B**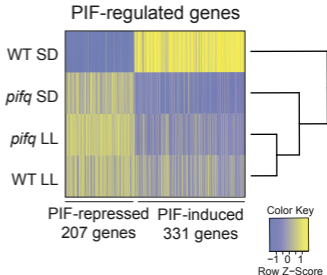

Supplement: Supplementary file 1 — Definition of 191 PIF/SD-induced and 145 PIF/SD-repressed genes. (A) Venn diagram showing the four-way comparison between SS 1.5F PIF-regulated (WT SD vs. pifq SD, induced and repressed) and SD-regulated (WT SD vs. WT LL, induced and repressed) gene sets. The number of differentially expressed genes in each set is indicated. The list of genes in each class is provided in Dataset S1. SS1.5F: genes whose expression changed statistically significantly and by at least 1.5 fold. (B) Two-dimensional-cluster diagram depicting the identified PIF- regulated SS1.5F genes in 3-day-old SD- and LL-grown WT and pifq seedlings. A total of 207 genes are upregulated (PIF-repressed) in the absence of PIFq, whereas 331 correspond to genes that are downregulated (PIF-induced), as defined in (A). [file Image_1.PDF]

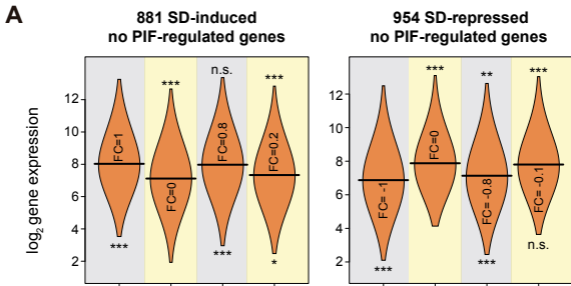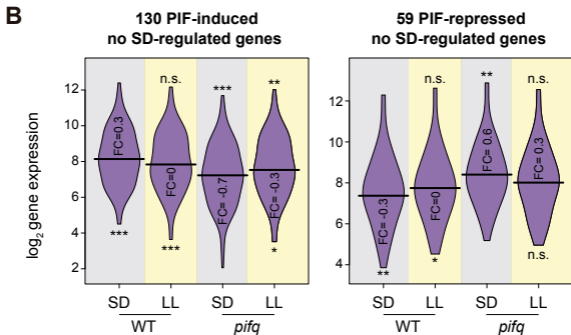

Supplement: FIGURE S2 — Gene expression of SS1.5F PIF-specific and SD-specific regulated genes. (A) Vioplots of log2 expression values in WT and pifq (SD and LL) of the 881 SD-induced (left) and the 954 SD-repressed (right) genes that are not regulated by PIFq, as defined in Supplementary Figure S1A. (B) Vioplots of log2 expression values in WT and pifq (SD and LL) of the 130 PIF-induced (left) and the 59 PIF-repressed (right) genes that are not regulated by SD, as defined in Supplementary Figure S1A. (A,B) Statistically significant differences from WT SD or WT LL by Willcoxon test are indicated in the upper and lower part, respectively (∗p-value < 0.05; ∗∗p-value < 0.01; ∗∗∗p-value < 0.001. n.s., non-significant). The mean log2 FC value relative to the expression value of WT LL for each sample is indicated. [file Image_2.PDF]

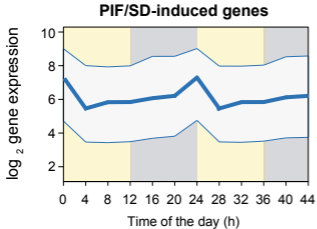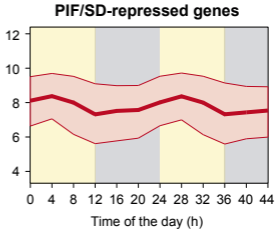

Supplement: FIGURE S4 — Diurnal pattern of PIF/SD-regulated genes in 12:12. Median values (thick line) and upper and lower quartiles (thin lines) of log2 expression for all PIF/SD-induced (left) and PIF/SD-repressed (right) under 12:12. Data were obtained from http://diurnal.mocklerlab.org. Day: yellow; Night: gray. [file Image_4.PDF]

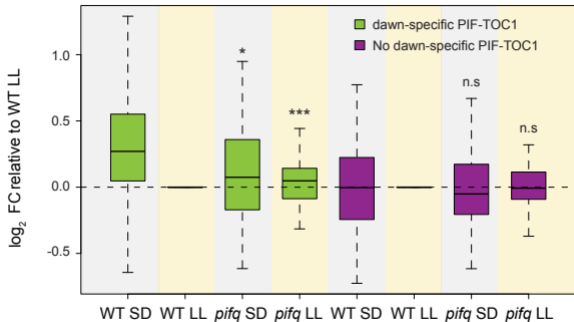

Supplement: FIGURE S6 — Expression of the previously defined “dawn-specific PIF-TOC1” and “no dawn-specific PIF-TOC1” in SD conditions. Box plots of microarray data showing the log2 FC expression in WT and pifq in SD and LL, relative to the WT LL, of the “dawn-specific PIF-TOC1” (green) and “no dawn-specific PIF-TOC1” (purple) genes recently defined by Soy et al. (2016). Statistically significant differences among FC values relative to WT SD by Willcoxon test are indicated (∗p-value < 0.05; ∗∗∗p-value < 0.001. n.s., non-significant). [file Image_6.PDF]
