# Supplementary material for: Genomic Analysis Reveals Contrasting PIFq Contribution to Diurnal Rhythmic Gene Expression in PIF-Induced and -Repressed Genes
Source: Front Plant Sci. 2016 Jul 4;7:962. doi: 10.3389/fpls.2016.00962 (PMC4930942; doi:10.3389/fpls.2016.00962)
Supplement: FIGURE S3 — PIF/SD-regulated genes ranked based on the percentage contribution of PIFq to their expression in SD. PIF/SD-induced (left) and PIF/SD-repressed genes (right) were arranged in descending percentage based on the contribution of PIFq to their expression in SD. Percentage of genes with a contribution of <50% or >100% is indicated for each gene set. [file Image_3.PDF]

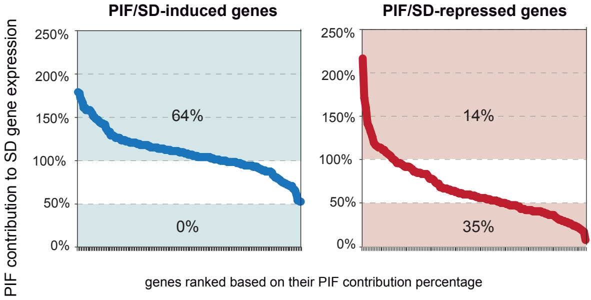

PIF/SD-induced genes
PIF/SD-repressed genes
PIF contribution to SD gene expression
250%
200%
150%
100%
50%
0%
64%
0%
14%
35%
genes ranked based on their PIF contribution percentage
