# Supplementary material for: Genomic Analysis Reveals Contrasting PIFq Contribution to Diurnal Rhythmic Gene Expression in PIF-Induced and -Repressed Genes
Source: Front Plant Sci. 2016 Jul 4;7:962. doi: 10.3389/fpls.2016.00962 (PMC4930942; doi:10.3389/fpls.2016.00962)
Supplement: FIGURE S5 — Expression of the SD-specific PIF/SD-regulated genes under the previously described shade, deetiolation and photoperiodic growth conditions. Bar graph of microarray data [deetiolation (Leivar et al., 2009), shade (Leivar et al., 2012), and photoperiodic conditions (Nozue et al., 2011)] showing the log2 FC expression relative to the WT Light in deetiolation and shade, and relative to pif4pif5 in growth phase, of the 76 PIF/SD-induced (top) and 63 PIF/SD-repressed (bottom) genes not previously defined as PIF-regulated in any of the other experimental conditions. SS, statistically significantly; FRed: Far red. [file Image_5.PDF]

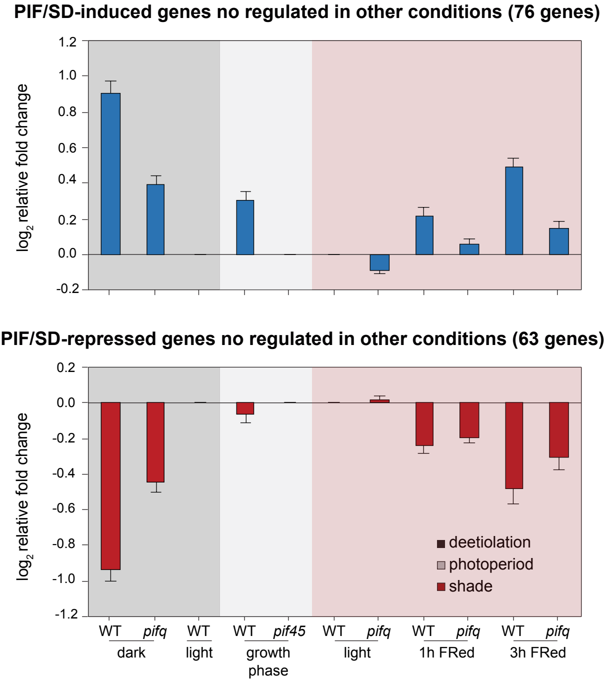
PIF/SD-induced genes no regulated in other conditions (76 genes)
log2 relative fold change
1.2
1.0
0.8
0.6
0.4
0.2
0.0
-0.2
PIF/SD-repressed genes no regulated in other conditions (63 genes)
0.2
0.0
-0.2
-0.4
-0.6
-0.8
-1.0
-1.2
deetiolation
photoperiod
shade
WT
pifq
WT
WT
pif45
WT
pifq
WT
pifq
WT
pifq
dark
light
growth phase
light
1h FRed
3h FRed
